# The contrasting roles of creep and stress relaxation in the time-dependent deformation during *in-situ* cooling of a nickel-base single crystal superalloy

**DOI:** 10.1038/s41598-017-10091-w

**Published:** 2017-09-11

**Authors:** Chinnapat Panwisawas, Neil D’Souza, David M. Collins, Ayan Bhowmik

**Affiliations:** 1 School of Metallurgy and Materials, University of Birmingham, Birmingham, B15 2TT United Kingdom; 2 Rolls-Royce plc, PO Box 31, Derby, DE24 8BJ United Kingdom; 3 Department of Materials, University of Oxford, Parks Road, Oxford, OX1 3PH United Kingdom; 4 Department of Materials, Imperial College London, Exhibition Road, London, SW7 2AZ United Kingdom

## Abstract

Time dependent plastic deformation in a single crystal nickel-base superalloy during cooling from casting relevant temperatures has been studied using a combination of *in-situ* neutron diffraction, transmission electron microscopy and modelling. Visco-plastic deformation during cooling was found to be dependent on the stress and constraints imposed to component contraction during cooling, which mechanistically comprises creep and stress relaxation. Creep results in progressive work hardening with dislocations shearing the γ′ precipitates, a high dislocation density in the γ channels and near the γ/γ′ interface and precipitate shearing. When macroscopic contraction is restricted, relaxation dominates. This leads to work softening from a decreased dislocation density and the presence of long segment stacking faults in γ phase. Changes in lattice strains occur to a similar magnitude in both the γ and γ′ phases during stress relaxation, while in creep there is no clear monotonic trend in lattice strain in the γ phase, but only a marginal increase in the γ′ precipitates. Using a visco-plastic law derived from *in-situ* experiments, the experimentally measured and calculated stresses during cooling show a good agreement when creep predominates. However, when stress relaxation dominates accounting for the decrease in dislocation density during cooling is essential.

## Introduction

The micromechanical deformation of single crystal nickel-base superalloys during cooling after solidification is not well understood. During cooling following solidification, progressive deformation of the solid metal occurs and the local cooling rate plays an important role in determining the local stress evolution. It is clear that any governing micromechanisms have their origins in high temperature deformation processes such as creep and stress relaxation^1–6^. Interestingly, creep experiments that have investigated mechanical deformation in certain stress and temperature conditions, replicate the environment experienced by the material during the investment casting process^7, 8^. Quantitative characterisation of high temperature deformation induced during casting of single crystal nickel-base superalloys is therefore vital to guide the design of future downstream processes, such as heat treatments, during which recrystallisation could occur.

Recrystallisation is a principal solidification defect in single crystal turbine blades; occurring from a build-up of stress due to localised strains that develop during cooling^7, 8^. Recrystallisation occurs during a subsequent solution heat treatment, where strain-free grains nucleate and grow from regions of high local strain (remnant plastic strain) into the matrix^7^. It is possible to calculate the stress and strain that develops during cooling using constitutive models, where both isotropic hardening as well as visco-plastic conditions could be considered with implications to the formation of recrystallised grains^8–11^. Such models implicitly assume a certain constitutive law where stress is considered as a function of time-dependent plastic strain rate, plastic strain and temperature. While it is accepted that visco-plasticity is primarily derived from mechanisms associated with creep, under certain conditions the role of stress relaxation must also be considered^12–14^. Identifying the micromechanisms that control such deformation requires the use of techniques that ideally possess both high spatial and angular resolution. Transmission electron microscopy (TEM) is one such method, however, observations are typically post-deformation and therefore do not reveal the temporal evolution of deformation or kinetic mechanisms, where the latter constitutes the critical basis for any analysis. If TEM is used in conjunction with a technique that permits time resolved measurements, such as neutron diffraction, this complimentary approach could offer sufficient information to elucidate the governing micro-mechanisms that may lead to recrystallisation. This approach also provides accurate data for numerical model validation used for the quantification of stresses and strains during casting.

In this paper, the evolution of the elastic strains in the γ matrix and γ′ precipitates have been evaluated *via* *in-situ* neutron diffractometry when subjecting single crystal nickel-base superalloy samples to the stresses and temperatures typically experienced following solidification during the casting process. It must be emphasised that while neutron diffraction is a potent method for the lattice (elastic) strain measurement at a constant temperature, cooling experiments are far from trivial^15^. This is because during cooling the signal is averaged over a range of temperatures dependant on the acquisition rate of the detector. For a smaller acquisition time, that is comparable to the local solidification time (*t* ~ 3 mins for a typical cooling rate of 1 °C min^-1^), the signal is acquired over a temperature range of approximately 3 °C, which is deemed to be sufficiently small. However, measurement accuracy is lost with a decreased signal to noise ratio associated with rapid data acquisition. A further aim of this study is to calculate the macroscopic stresses and strains that develop in an incremental temperature interval during cooling *via* a numerical modelling approach. To this end an expression for the inelastic strain is required, which is either isotropic hardening (*i.e*. without invoking any specific slip system) or a visco-plastic strain rate, when rate-dependent deformation occurs. In this study, we conduct a series of creep and stress relaxation experiments over a range of temperatures and stresses to derive such a visco-plastic law. In doing so we critically assess the basis for deriving such an equation in relation to similar approaches adopted by others^7–9^. The experimental parameters are usually obtained from curve-fitting of data generated from steady-state creep experiments conducted over several hours. This study uses experimental data collected over a time interval typical of the local solidification time in a different manner. Importantly, we also consider the role of stress relaxation, since both stress relaxation and creep occur simultaneously. This enables one to identify the conditions under which each of the two visco-plasticity phenomena dominate. In order to examine the mechanical response of γ and γ′ phases during deformation, the respective microstrains that develop within the individual phases are separated. It has been demonstrated that significant strain partitioning occurs between γ and γ′ phases depending on temperature, applied stress and deformation rate^13, 16–18^. Further, from a detailed examination of the underlying dislocation structure we identify the associated micromechanisms when creep and stress relaxation dominate. This aspect is specifically lacking in previous studies, with only limited evidence of the development of dislocation structure at temperatures close to the solvus temperature^19^. It should be emphasised that this aspect is critical for establishing a reliable materials database in terms of plastic strain and its correspondence to dislocation density^20^.

## Method

### *In-situ* Neutron Diffraction

Uniaxial as-cast tensile specimens of CMSX-4 were manufactured *via* the investment casting route, after^14^. The specimens having diameter 5.9 mm and gauge length 29 mm were directionally solidified as single crystals. To ensure that the axial orientation of the specimens was within 5° from 〈001〉, the specimens were seeded. Details of the method can be obtained from^21^. The nominal composition of CMSX-4 is given in Table 1. *In-situ* experiments have been carried out on the ENGIN-X beamline at the ISIS Neutron and Muon Source, Rutherford Appleton Laboratory, Didcot, UK. An optical furnace was used to heat the samples in air and a K-type thermocouple was held in contact with the sample to monitor temperature profiles. The loading axis in these experiments was horizontal, at 45° to the incident beam, with the samples mounted with the applied stress parallel to the specimen axis. The detectors were fixed at 90° to the incident beam and the data was obtained from the full ±15° detector bank. The measurement of lattice spacing was restricted to the longitudinal direction, *i.e*. specimen axis^14–16, 18^. Heating was carried out at 10 °C min^−1^ from room temperature to 800 °C and then at 5 °C min^−1^ to the set temperature under zero load to allow for thermal expansion. Samples were held at the set temperature for 9 mins to allow thermal equilibration to be reached. Load was then applied at 0.2% min^−1^ until a target macroscopic stress was reached and held at that load for an additional 9 mins. Thereafter, either cooling tests or isothermal stress relaxation and creep tests were carried out. The pattern acquisition time was 3 mins throughout the entire heating, isothermal hold and cooling period. The selected time interval of 3 mins was deemed representative of the local cooling rate under typical casting conditions. Neutron diffraction measurements were used to obtain inter-planar spacings and the corresponding lattice strains that develop.

**Table 1:** The nominal composition (in weight %) of CMSX-4 single-crystal superalloy.

Cr	Co	Mo	Re	W	Al	Ti	Ta	Hf	Ni
6.5	9	0.6	3	6	5.6	1	6.5	0.1	Balance

### Cooling Tests

Cooling experiments were performed for two different initial stresses of 280 MPa and 350 MPa – these two experiments are hereafter referred to as ‘expt. 1’ and ‘expt. 2’ respectively and commencing from a starting temperature of 1000 °C. Cooling was carried out at a nominal rate of 1 °C min^−1^ down to 950 °C. During cooling, the sample was held under strain control to replicate the strain development following solidification, where the contraction of the metal is restrained by a ceramic core thereby placing the metal in tension. The initially applied stresses of 350 MPa and 280 MPa are within the range of stresses known to develop in the metal during solidification at 1000 °C^8, 9^.

### Isothermal Tests (Creep and Stress Relaxation)

In isothermal tests, creep and stress relaxation experiments were carried out at two representative temperatures of 950 °C and 1000 °C over a wide range of stresses. The initial stresses were 340 MPa and 280 MPa respectively, with an increment of 10 MPa. Following application of load and during subsequent dwell under constant load for 9 mins the sample creeps with strain continuously measured by an extensometer attached to the specimen. Thereafter, a stress relaxation test under strain control (fixed ends of the sample) was carried out for an additional 12 mins. The measured lattice strain corresponded to that of the composite (200)_γ + γ′_ peak^14, 15^. The strain is reported as a function of temperature and stress normalised by the 0.2% yield stress, ${\sigma }_{0}$, *i.e.* $\sigma /{\sigma }_{0}$.

### Micromechanics

The neutron diffraction reflections measured were each fitted with an asymmetric line profile, comprising a convolution product, $\otimes$, between a Voigt function, $V(x)$, and an exponential function. The independent variable is defined as $x=d-{d}_{c}\,\,$where $d$ is the *d*-spacing and ${d}_{c}$ is the centre of mass of the reflection to be fitted. The fitted function is given by

1 $${V}_{\exp }(x)=V(x)\otimes \exp (\eta x)H(x)$$

where $\eta$ is a constant dependent on *d*, obtained from fitting all available reflections measured in a CeO_2_ standard with the *d*-spacing range 1.19 Å < *d* < 2.88 Å. $H(x)$ is the Heaviside step function. The γ′ lattice parameter, ${a}_{{\rm{\gamma }}}$, was calculated from the fitted positions of the (100)_γ′_ and (300)_γ′_ superlattice reflections. The line profile position of the (200)_γ′_ reflection, ${d}_{(200)}^{{\gamma }^{^{\prime} }}$, can then be inferred^22^. The γ lattice parameter, ${a}_{{\rm{\gamma }}}$ was obtained from fitting the (200) fundamental reflection, accounting for the superposition of intensity from the γ and γ′ phases. Two line profiles were fitted; one with ${d}_{(200)}^{{\gamma }^{^{\prime} }}$ fixed and one with ${d}_{(200)}^{{\rm{\gamma }}}$, the γ line profile position, allowed to vary. Obtaining the correct value of ${d}_{(200)}^{{\rm{\gamma }}}$ has been the subject of prior investigations, detailed in^18^ and^23^. The method demonstrates that the ${d}_{(200)}^{{\rm{\gamma }}}$ is only obtained when the correct ratio of intensity between the γ and γ′ phases has been found. This is dependent on the respective phase volume fractions and composition dependent structure factors; this study obtained these values from the thermodynamic software, JMatPro^TM^ ^24^. These calculated values were within good agreement of the volume fraction values for CMSX-4 obtained by Roebuck, B. *et al*.^25^. For full details of the fitting procedure and the refinement process applied to neutron diffraction, the reader is referred to^18^.

Using the measured values of ${d}_{(200)}^{{\rm{\gamma }}}\,$and ${d}_{(200)}^{{\gamma }^{^{\prime} }}$, lattice strains were calculated for data obtained during sample cooling. The lattice strains in the[100] direction, ${\varepsilon }_{(200)}^{{\rm{\gamma }}}$ and ${\varepsilon }_{(200)}^{{\gamma }^{^{\prime} }}\,\,$were calculated using

2 $${({\varepsilon }_{(200)}^{\gamma ,{\gamma }^{^{\prime} }})}_{T}={(\frac{{d}_{(200)}^{\gamma ,{\gamma }^{^{\prime} }}-{d}_{(200)}^{\gamma ,{\gamma }^{^{\prime} }}(\sigma =0)}{{d}_{(200)}^{\gamma ,{\gamma }^{^{\prime} }}(\sigma =0)})}_{T}$$

where ${d}_{(200)}^{\gamma ,{\gamma }^{^{\prime} }}(\sigma =0)$ is the relaxed value of *d*-spacing for the γ or γ′ phase when no stress is applied for a given temperature, $T.$ Values of ${d}_{(200)}^{\gamma ,{\gamma }^{^{\prime} }}(\sigma =0)$ were obtained from the fitted line profile positions for the data collected during heating. A polynomial function was fitted to ${d}_{(200)}^{\gamma ,{\gamma }^{^{\prime} }}(\sigma =0)$ values as a function of temperature. This function was subsequently used to estimate ${d}_{(200)}^{\gamma ,{\gamma }^{^{\prime} }}(\sigma =0)$ for the corresponding cooling temperature.

### Transmission Electron Microscopy

Specimens for transmission electron microscopy (TEM) were prepared from the tensile bar gauge section by electro-discharge machining discs of 3 mm diameter. Approximately 3–4 specimens were prepared from the gauge in each sample, each ground to ~200–250 μm thickness. Foils were produced from the disc specimens *via* electro-polishing using a solution of 10 vol.% perchloric acid and 90 vol.% methanol at −40 °C. Given that each tensile bar was orientated with its axial direction within 5° from the [001] direction, each TEM foil normal was close to the [001] direction. Foils were examined using a double-tilt specimen holder in a JEOL 2100 Plus transmission electron microscope at an accelerating voltage of 200 kV. Using various tilts, bright field (BF) and weak beam dark field (WBDF) (g-3g condition) images were obtained to observe the dislocation structures in each sample. In order to identify the nature of dislocations shearing the γ′ precipitates, a series of bright field images were recorded from several two-beam conditions using reflections from three zone axes found close to the foil normal, *i.e*. [001], [114] and [112]. Images were obtained using four diffraction vectors, $\bar{2}00$, $\bar{2}\bar{2}0$, $0\bar{2}0$ and $2\bar{2}0$, from the [001] zone axis, one vector, $31\bar{1}$, from the ﻿[114] zone axis and two vectors, $1\bar{3}1$ and $3\bar{1}\bar{1}$, from the ﻿[112] zone axis.

### Modelling approach

In order to simulate the evolution of stress and strain during cooling from high temperature, uniaxial tension under strain control has been modelled by assuming the decomposition of strain as:^8, 9^

3 $${\varepsilon }^{{\rm{total}}}=\,{\varepsilon }^{{\rm{el}}}+\,{\varepsilon }^{{\rm{vp}}}+\,{\varepsilon }^{{\rm{th}}}$$

which follows the linear elasticity law:

4 $$\sigma =E{\varepsilon }^{{\rm{el}}}$$

Here, ${\varepsilon }^{{\rm{total}}}$, ${\varepsilon }^{{\rm{el}}}$, ${\varepsilon }^{{\rm{vp}}}$, ${\varepsilon }^{{\rm{th}}},$ $\sigma$ and $E$ are total strain, elastic strain, visco-plastic (time-dependent) strain, thermal strain, effective stress and Young’s modulus respectively. The thermal strain is described as; ${\varepsilon }^{{\rm{th}}}(T)=\,\alpha (T-{T}_{{\rm{ref}}})$, where $\alpha$ is thermal expansion coefficient, $T$ is temperature and ${T}_{{\rm{ref}}}$ is reference temperature. The constitutive behaviour of visco-plasticity has been considered to comprise of two contributions, *i.e*. relaxation softening and creep hardening,

5 $${\dot{\varepsilon }}^{{\rm{vp}}}(T,\sigma ,\varepsilon )=\,{\dot{\varepsilon }}^{{\rm{rel}}}(T,\sigma ,\varepsilon )+\,{\dot{\varepsilon }}^{{\rm{cr}}}(T,\sigma ,\varepsilon )$$

where, ${\dot{\varepsilon }}^{{\rm{vp}}}=\,\frac{{\rm{d}}{\varepsilon }^{{\rm{vp}}}}{{\rm{d}}t}$, ${\dot{\varepsilon }}^{{\rm{rel}}}=\,\frac{{\rm{d}}{\varepsilon }^{{\rm{rel}}}}{{\rm{d}}t}$ and ${\dot{\varepsilon }}^{{\rm{cr}}}=\,\frac{{\rm{d}}{\varepsilon }^{{\rm{cr}}}}{{\rm{d}}t}$ are visco-plastic strain rate, relaxation strain rate and creep strain rate, respectively. Relaxation rate and creep rate follow the Arrhenius type relations, which can be defined as:^15^

6 $${\dot{\varepsilon }}^{{\rm{rel}}}=\,{A}_{0}^{{\rm{rel}}}\exp (-\frac{{Q}^{{\rm{rel}}}}{RT})\,{(\frac{\sigma }{{\sigma }_{0}})}^{{n}^{{\rm{rel}}}}$$

7 $${\dot{\varepsilon }}^{{\rm{cr}}}=\,{A}_{0}^{{\rm{cr}}}\exp (-\frac{{Q}^{{\rm{cr}}}}{RT})\,{(\frac{\sigma }{{\sigma }_{0}})}^{{n}^{{\rm{cr}}}}$$

Here ${A}_{0}^{{\rm{rel}}}$, ${A}_{0}^{{\rm{cr}}}$, ${Q}^{{\rm{rel}}}$, ${Q}^{{\rm{cr}}}$, ${n}^{{\rm{rel}}}$, ${n}^{{\rm{cr}}}$, $R$ and ${\sigma }_{0}$ are relaxation pre-exponent, creep pre-exponent, relaxation activation energy, creep activation energy, relaxation exponent, creep exponent, molar gas constant and yield stress, respectively. In this study, the parameters in Equations (6) and (7) are obtained from curve fitting of data from *in-situ* isothermal tests using neutron diffraction and the extensometer measured strain respectively at temperatures 950 °C and 1000 °C. For the cooling experiments, the cooling rate, $\dot{T}=\,\frac{{\rm{d}}T}{{\rm{d}}t}$, used in this work is nominally 1 °C min^−1^ (0.0﻿17 °C s^−1^). Two different initial stresses, ${\sigma }_{{\rm{i}}}$, *i.e*. 280 MPa and 350 MPa, which are below and just above the yield stress at 1000 °C (325 MPa) are applied at 1000 °C to simulate the evolution of stress and time-dependent plastic strain during cooling between 1000 °C to 950 °C.

A three-dimensional (3D) model using the commercial software package ABAQUS/Standard was used to calculate the evolution of stress in the as-cast tensile test bar during cooling. To simulate the mechanical deformation during the *in-situ* cooling experiments, a 3D tensile bar geometry was generated and replicates the cooling at the applied stresses of 280 MPa and 350 MPa. Using the available elasto-plastic model with isotropic hardening:^8, 9^

8a $$\sigma =\,{\sigma }_{{\rm{UTS}}}+\,({\sigma }_{{\rm{UTS}}}-\,{\sigma }_{0})\exp (-h{\varepsilon }^{{\rm{pl}}}),$$

where, ${\sigma }_{{\rm{UTS}}}=\mathrm{896}\,\mathrm{MPa}\,\mathrm{at}\,\mathrm{900}$ °C ${\rm{and}}\,{\rm{757}}\,{\rm{MPa}}\,{\rm{at}}\,{\rm{1000}}$ °C, and $h=0.3$ are ultimate tensile strength and hardening coefficient respectively. The von Mises stress, ${\sigma }_{{\rm{v}}}$ is calculated and defined as

8b $${\sigma }_{{\rm{v}}}=\,\sqrt{\frac{1}{2}[{({\sigma }_{11}-{\sigma }_{22})}^{2}+\,{({\sigma }_{22}-{\sigma }_{33})}^{2}+\,{({\sigma }_{33}-{\sigma }_{11})}^{2}+6({\sigma }_{12}^{2}+{\sigma }_{23}^{2}+{\sigma }_{31}^{2})]}$$

where, ${\boldsymbol{\sigma }}=\,({\sigma }_{11},{\sigma }_{22},\,{\sigma }_{33},{\sigma }_{12},{\sigma }_{31},{\sigma }_{23})$ is a stress tensor. The elasto-plastic law was adopted from the available data at high temperatures^9^.

In the case of a simple one-dimensional (1D) model, according to Equation (3), the stress and strain development during cooling can be described as follows:^15^

9 $$\alpha {\rm{\Delta }}T-{\rm{\Delta }}\varepsilon =\frac{{\rm{\Delta }}\sigma }{E}+{\int }_{0}^{{\rm{\Delta }}t}\frac{{\rm{d}}{\varepsilon }^{{\rm{vp}}}}{{\rm{d}}t}{\rm{d}}t$$

where Δ*ε* is the change in strain during cooling from $T$ to $[T\,-\,{\rm{\Delta }}T]$ in time interval $t\,(T\, > \,0)$. In the case of stress free contraction, ${\rm{\Delta }}\varepsilon \,=\,\alpha {\rm{\Delta }}T$, *i.e*. Δ*σ* = 0, in the case of complete constraint $\,{\rm{\Delta }}\varepsilon =\,0$ and for all other cases, $\,{\rm{\Delta }}\varepsilon  > \,0$. The numerical result is then compared with the experimental data.

## Results

### Evolution of lattice strain and stress during cooling

**Figure 1:**
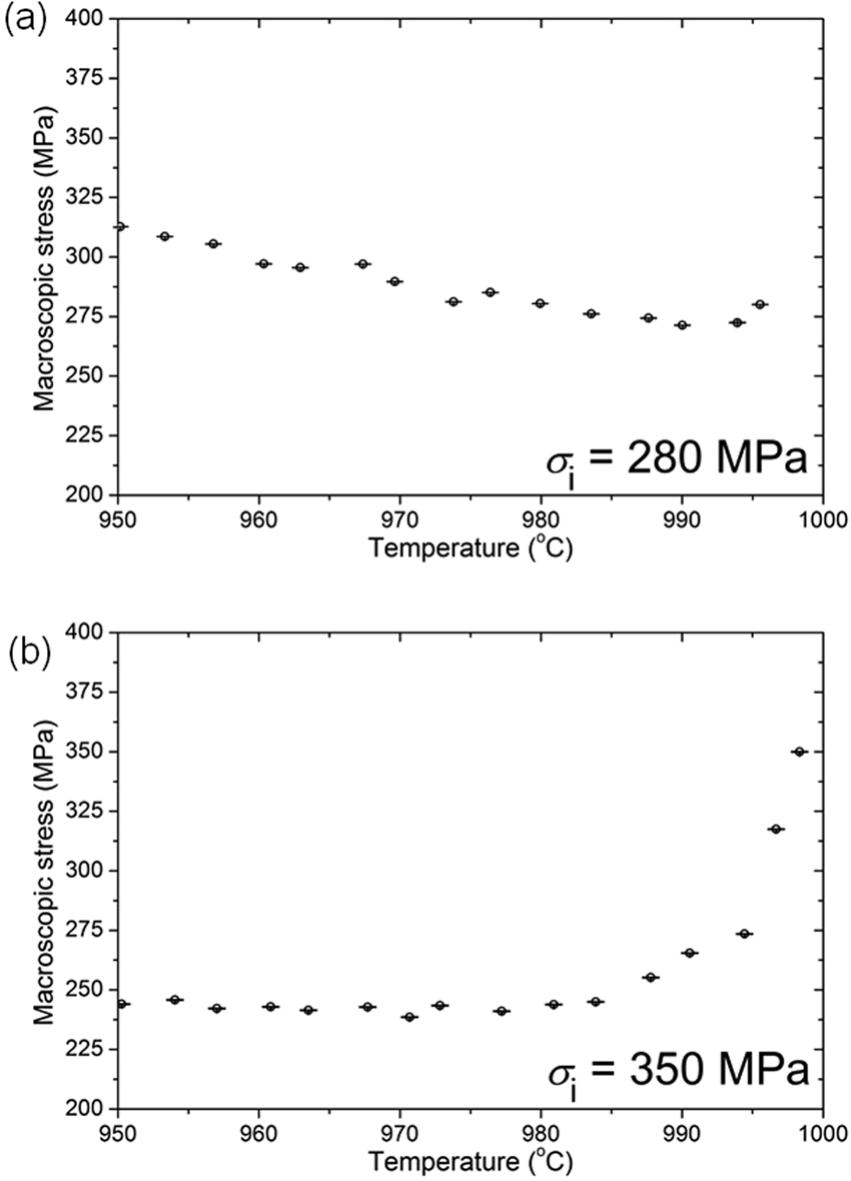
Experimentally measured stress during continuous cooling from the initial stresses; (**a**) 280 MPa, (**b**) 350 MPa.

Figure 1 presents the evolution of macroscopic stress with temperature during continuous cooling at a nominal cooling rate of 1 °C min^−1^, where the initial stresses were, ${\sigma }_{{\rm{i}}}$ = 280 MPa (expt. 1) and 350 MPa (expt. 2) respectively. In expt. 1, an initial stress relaxation of 8 MPa was observed in the temperature range, 994 °C < T ≤ 996 °C and thereafter the stress increases continuously up to 950 °C. In expt. 2, the initial stages of cooling were accompanied by a decrease in stress of 105 MPa within 15 mins and corresponding to the temperature range, 984 °C < T ≤ 998 °C. On further cooling the stress remains nearly constant over the temperature range, 950 °C ≤ T ≤ 984 °C.

**Figure 2:**
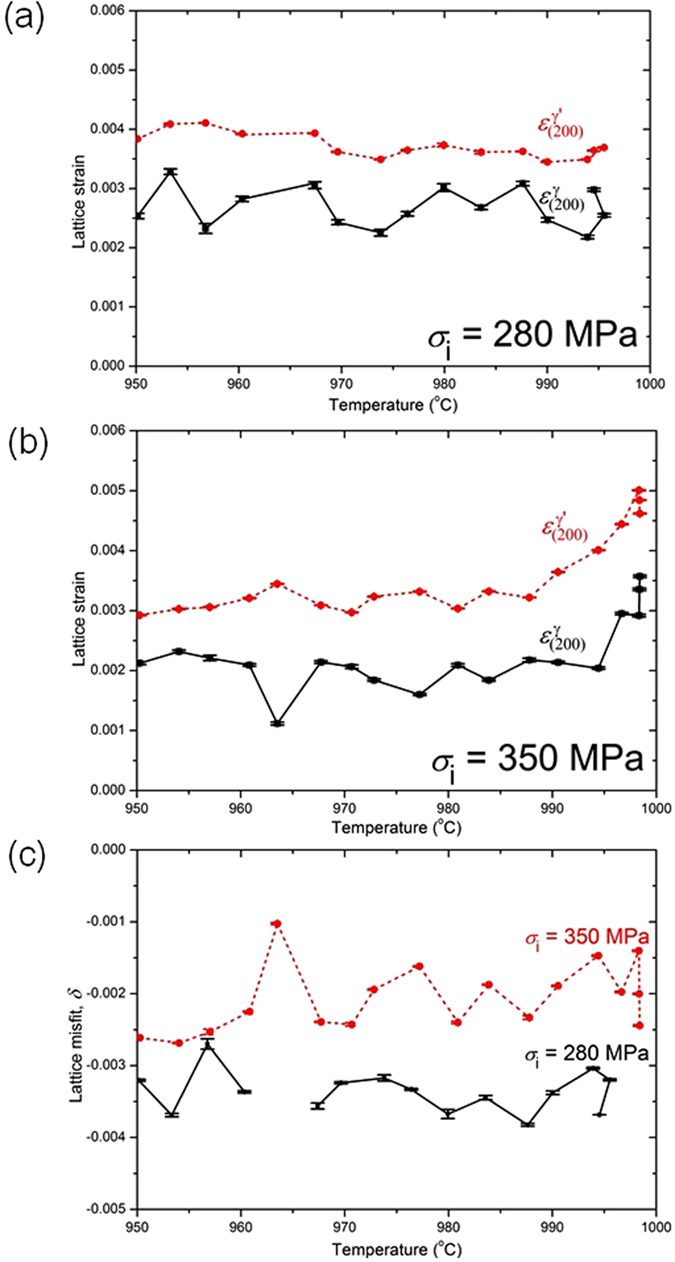
Lattice strains in γ and γ^′^ during continuous cooling for initial stresses at (**a**) 280 MPa and (**b**) 350 MPa. The variation of lattice misfit with temperature for these different initial stresses is shown in (**c**).

The evolution of lattice strains within the γ and γ′ phases corresponding to expts. 1 and 2 is presented in Figures﻿ 2(a and b) respectively, while the lattice misfit, $\delta =2({a}_{(200)}^{{\gamma }^{^{\prime} }}-\,{a}_{(200)}^{\gamma })/({a}_{(200)}^{{\gamma }^{^{\prime} }}+\,{a}_{(200)}^{\gamma })$ for both cases shown in Figure  2(c). In expt. 2, stress relaxation is prominent in 984 °C < T ≤ 998.5 °C, where the γ′ lattice strain, ${\varepsilon }_{(200)}^{{\gamma }^{^{\prime} }}$, decreases from 5 × 10^−3^ to 3.3 × 10^−3^, *i.e*. $\,{\rm{\Delta }}{\varepsilon }^{{\rm{el}}}$ = − 1.7 × 10^−3^ and γ lattice strain, ${\varepsilon }_{(200)}^{\gamma }$, decreases from 2.9 × 10^−3^ to 1.8 × 10^−3^, *i.e*. ${\rm{\Delta }}{\varepsilon }^{{\rm{el}}}$ = − 1.1 × 10^−3^. In expt. 1, a small amount of stress relaxation is observed in the range 994 °C < T ≤ 996 °C, where ${\varepsilon }_{(200)}^{{\gamma }^{^{\prime} }}$ decreases from 3.7 × 10^−3^ to 3.5 × 10^−3^, *i.e*. ${\rm{\Delta }}{\varepsilon }^{{\rm{el}}}$ = −0.2 × 10^−3^ and ${\varepsilon }_{(200)}^{\gamma }$ decreases from 2.6 × 10^−3^ to 2.2 × 10^−3^, *i.e*. $\,{\rm{\Delta }}{\varepsilon }^{{\rm{el}}}$ = −0.4 × 10^−3^. In general, the lattice strains follow the variations in the macroscopic stress. It must also be emphasised that the misfit in the lattice parameter between γ′and γ is negative, *i.e*. ${a}_{(200)}^{{\gamma }^{^{\prime} }}\, < \,{a}_{(200)}^{\gamma }$.

The dominant macroscopic stress relaxation during initial cooling is then followed by either a near-constant or increasing stress during subsequent cooling in both experiments. In expt. 2, a near-constant stress during subsequent cooling up to 950 °C indicates that thermal strain must be almost primarily accommodated through plastic deformation. This is because Δ*σ* ≈ 0 and under strain control, Δε = 0 and therefore from Equation 9 it follows that there is a perfect balance between hardening and recovery. This primarily arises from consideration of stress, since the applied stress just exceeds the yield stress (σ_0_ = 325 MPa). While in expt. 1, there is an increase in stress during subsequent cooling up to 950 °C that indicates that either (i) the deformation is entirely elastic and hence all the thermal strain is accommodated elastically or (ii) plastic deformation also occurs and is accompanied by work hardening resulting in an increase in stress. In the cooling cycle, 950 °C < T ≤ 994 °C, the ${\varepsilon }_{(200)}^{\gamma }$ fluctuates by, $\,{\rm{\Delta }}{\varepsilon }^{{\rm{el}}}$ ~ ± 0.5 × 10^−3^, while ${\varepsilon }_{(200)}^{{\gamma }^{^{\prime} }}\,\,$shows a marginal increase, $\,{\rm{\Delta }}{\varepsilon }^{{\rm{el}}}$ ~ 0.2 × 10^−3^.

### Deformation Microstructures Observed during Cooling

**Figure 3:**
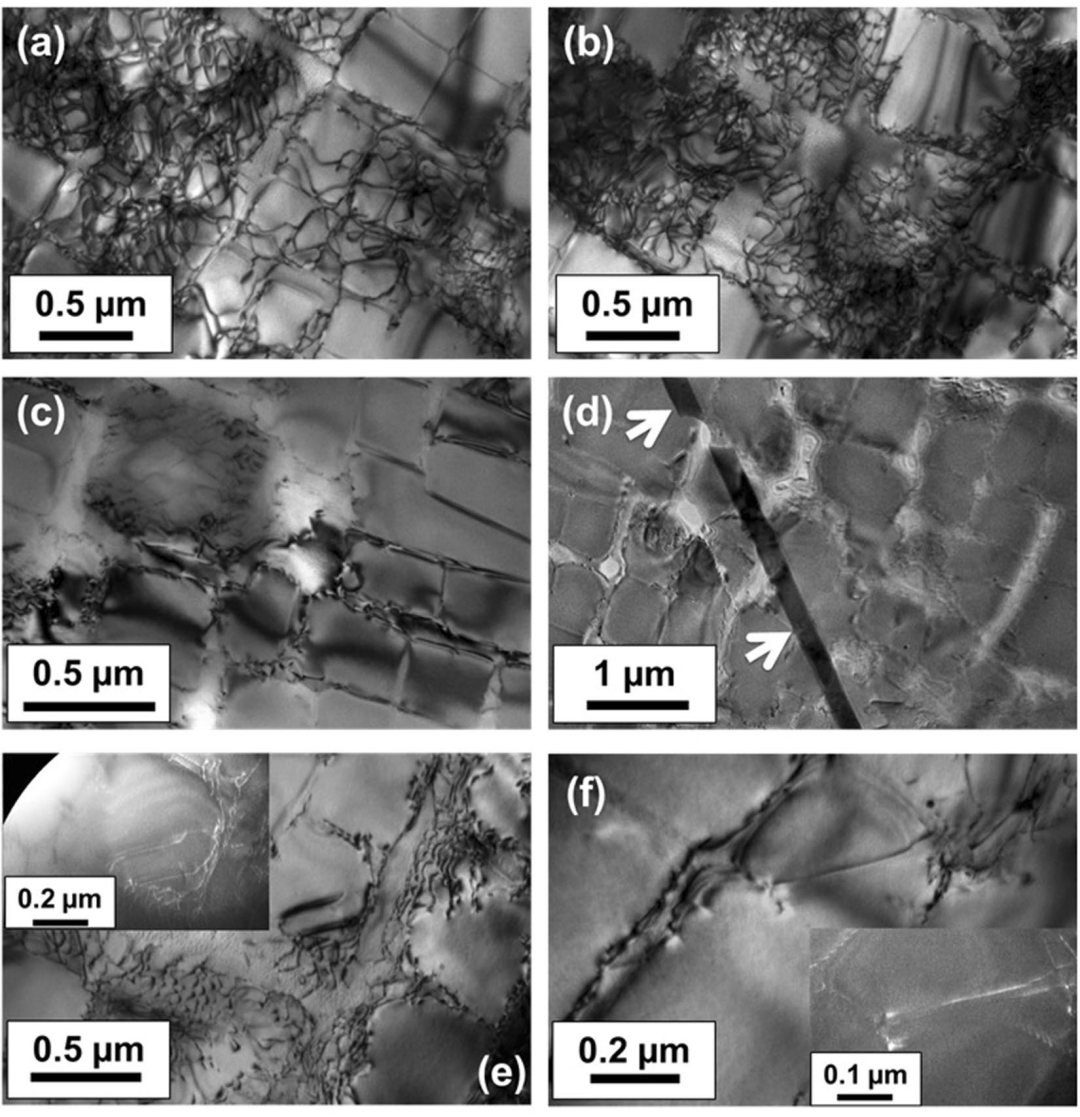
(**a**,**b**) Dislocation structure at low magnification in the cooled specimen with 350 MPa applied stress. The specimen was observed in bright field mode showing a high density of interfacial dislocation networks, imaged near the [001]-zone axis. (**c**) Large sections of the microstructure were, however, free of dislocations. (**d**) A stacking fault (indicated with white arrow) was also observed in the specimen foil in the γ channel cutting across the γ′ -precipitates. Some dislocations were found to shear the γ′-precipitates (**e** and **f**) which in the weak-beam dark field mode were observed to be closely spaced partial dislocations, shown in the insets of (**e**) and (**f**).

Figure 3 shows various regions of the microstructure in expt. 2 following relaxation. The micrographs were obtained close to the [001] zone axis using either ***g*** = 200 or 220 reflections. It is noted that in general, a low dislocation density was observed inside the horizontal and vertical channels of the γ phase [Figure  3(c)]. Under these viewing conditions, the sample however showed some regions with a high density of interfacial dislocation networks at the γ/γ′ interface [Figure﻿s 3(a and b)]. Such interfacial dislocations, present either as rows or networks, were wrapped around the surface of the cuboidal γ′ precipitates. The sample also showed in some instances the presence of stacking faults (length > 5 µm) in the γ phase and traversing across several precipitates [Figure  3(d)]. The γ′ precipitates primarily showed a defect-free structure with only a very small fraction of them sheared by dislocations. A closer look at the sheared γ′ precipitates revealed the dislocations were present as pairs and dipoles when imaged under both bright field and weak beam dark field (***g*** = 200) modes [Figure﻿s 3(e and f)]. The nature of these dislocations in γ′ has been discussed subsequently. However, no dislocation loops were observed in the sample.

**Figure 4:**
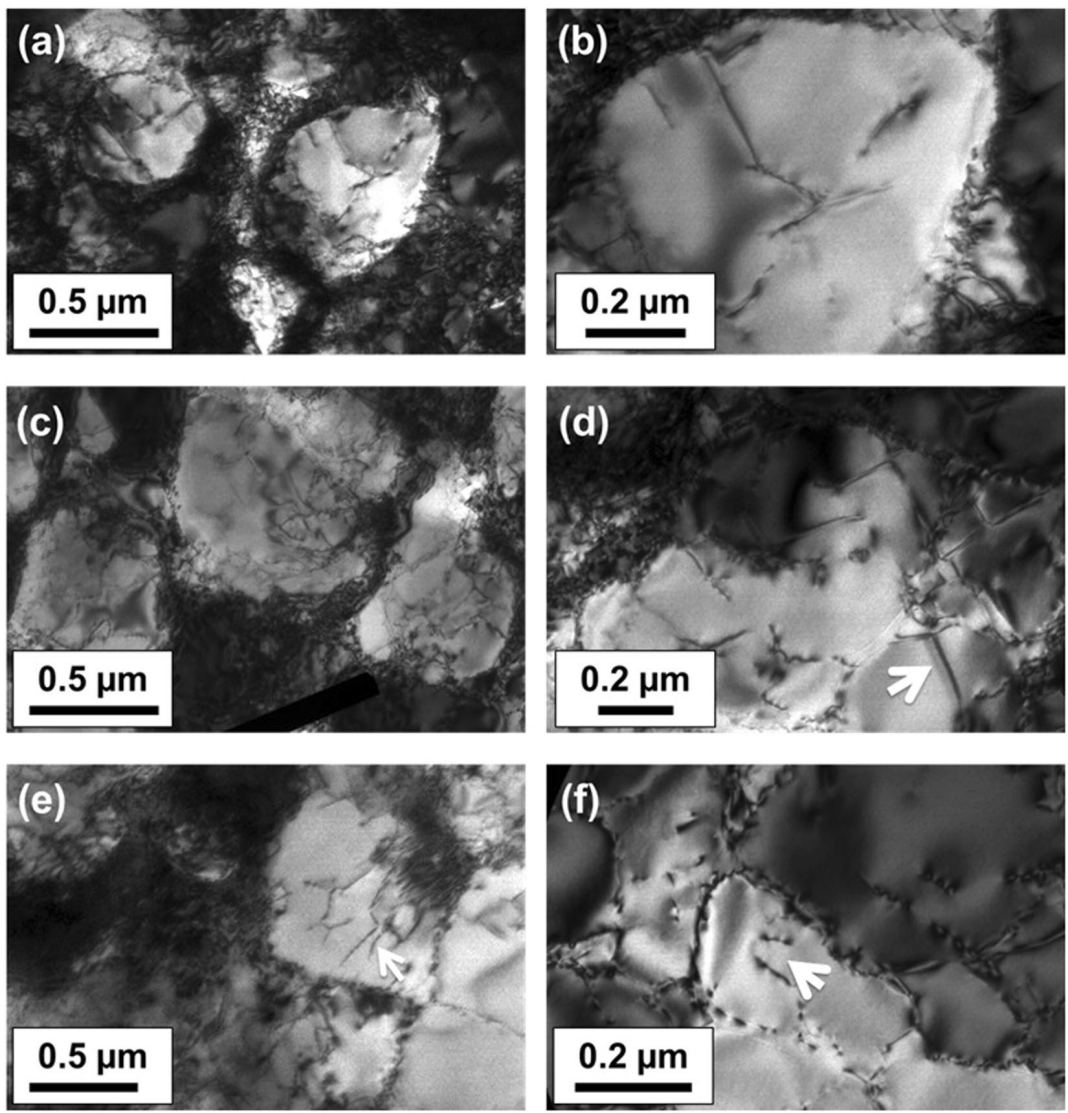
(**a**–**d**) Dislocation structure observed in the cooled specimen with 280 MPa applied stress with a very high density of dislocations in the γ channels, a high density network of dislocations near the γ/γ′ interface and also inside the γ′ precipitates. Precipitate shearing by dislocations was observed (**e** and **f**) – the shearing dislocations are either pairs of partials (shown by white arrow in (**d**)) or dipoles (shown by arrows in (**e**) and (**f**)).

Figure 4 shows the dislocation structure in expt. 1. Clearly a high degree of strain contrast is visible in the sample as compared to expt. 2. A high dislocation density was generally observed both in the γ channels and localised near the γ/γ′ interface [Figure﻿s 4(a–c)]. The high density of dislocations near the γ/γ′ interface blurred the outlines of the interfaces and thereby limiting the observation of the misfit interfacial dislocations. However, the interfacial dislocations were only observed in certain regions of the sample and under specific viewing conditions. The γ′ precipitates interestingly were found to undergo significant shearing by dislocations. The dislocations in the γ′ appeared as single dislocations or dipoles [Figure﻿s 4(d–f)]. No stacking faults or dislocation loops were observed in the sample. In summary, it is clear that there exists a greater overall dislocation density observed in the micrographs corresponding to expt. 1 compared to expt. 2.

**Figure 5:**
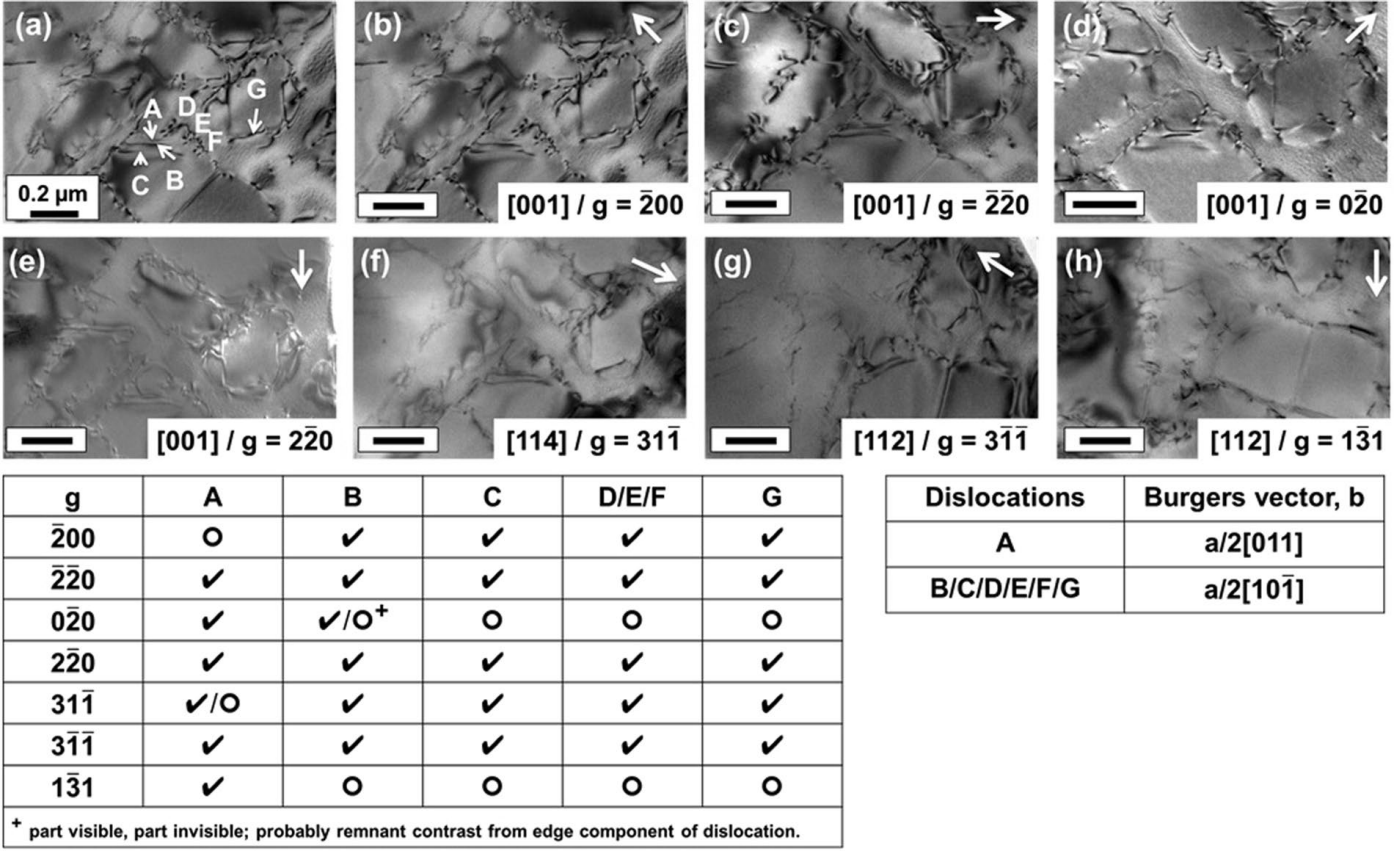
Dislocation Burgers﻿ vector analysis with dislocations A–G imaged under various two-beam conditions in the cooled specimen with 350 MPa applied stress. The scale bars on each image measure 0.2 µm.

In order to study the nature of the dislocations shearing the few γ′ precipitates at a given stress state in expt. 2, the Burgers vector of the dislocations was obtained using a series of two-beam conditions. The region of interest shown in Figure  5(a) was imaged under different two-beam conditions obtained from [001] in Figure﻿s 5(b–e), [114] in Figure  5(f) and [112] in Figure﻿s 5(g and h) zone axes. This region was interesting to observe since it showed some dislocation pairs in γ′ already having sheared the precipitate (dislocations A, B, C, G) and some dipoles just about to enter the γ′ precipitate (D, E and F) from the matrix phase. The table in Figure  5 summarises the results and outlines the reflections that were used for the two-beam analyses from the three zone axes to image the dislocations and under conditions the dislocations were in (visible, ✓) and out of (invisible, ○) contrast. Clearly, dislocations B to G showed similar contrast under the two-beam conditions while dislocation A behaved differently. Dislocation A was invisible under ***g*** = $\bar{2}00$ and $31\bar{1}$ and visible under remaining conditions which implied it had a Burgers vector of $\frac{a}{2}[011]$. On the other hand, dislocations B to G were determined to have a Burgers vector of $\frac{a}{2}[10\bar{1}]$ since they were invisible under ***g*** = $0\bar{2}0$ and $1\bar{3}1$ conditions. In the image obtained with ***g*** = $0\bar{2}0$, while a part of it is out of contrast, some part of dislocation line B is found to be visible – this contrast is the residual contrast that is typically observed in edge dislocations owing to atomic distortion near the core of the dislocation, although some local bending of the TEM foil should also possibly be considered.

### Isothermal Creep and Stress Relaxation Experiments

**Figure 6:**
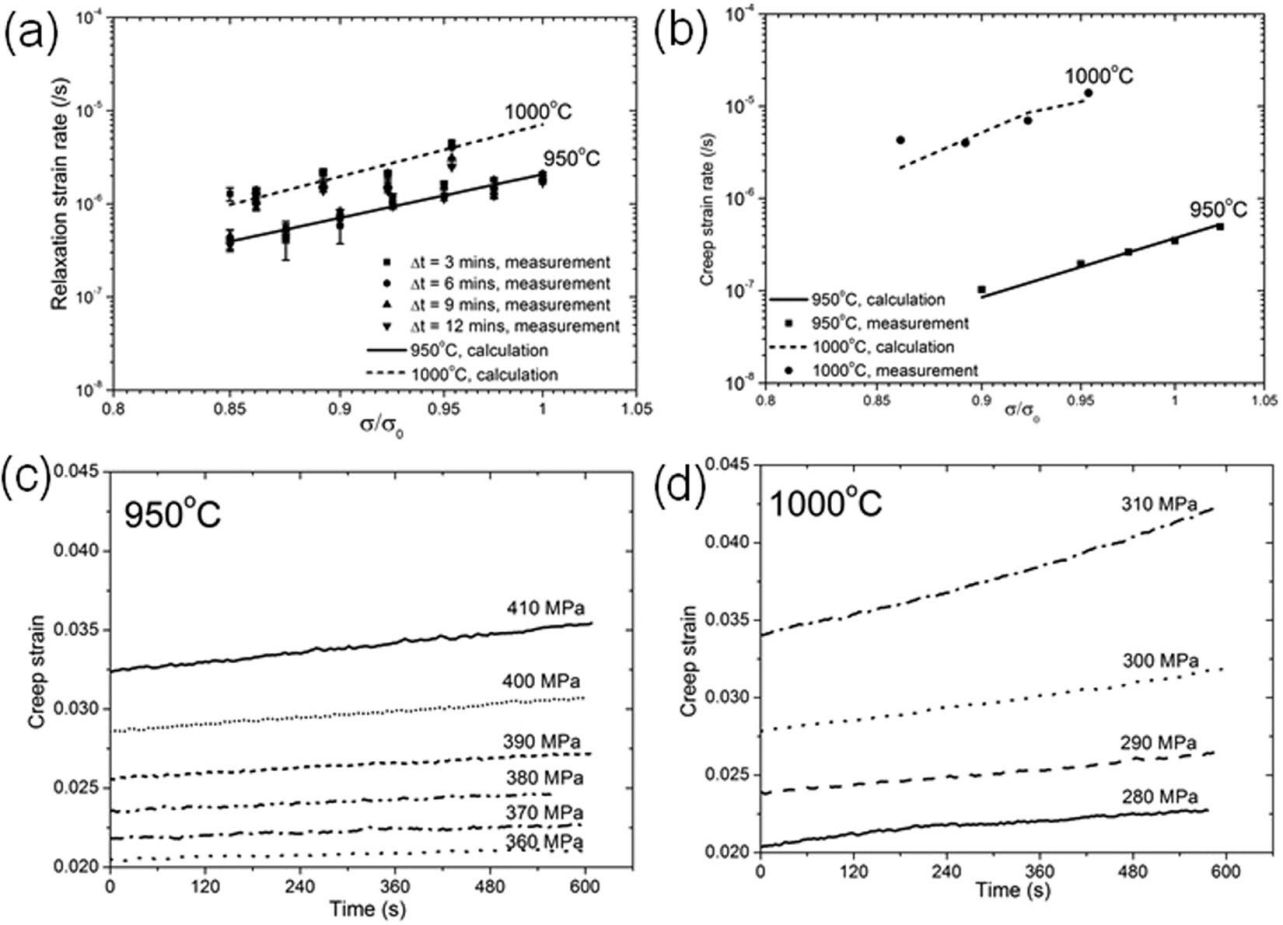
Time dependent strain rates during isothermal loading; (**a**) relaxation strain rate and (**b**) creep strain rate at 950 °C and 1000 °C; and creep strain over 9 min holding at isothermal testing at (**c**) 950 °C and (**d**) 1000 °C.

Figure 6(a) presents the evolution of elastic strain rate with $\sigma /{\sigma }_{0}$ at temperatures of 950 °C and 1000 °C respectively. It can be observed that at each temperature and for any given time, the extent of stress relaxation increases with $\sigma /{\sigma }_{0}$. At 1000 °C and for $\sigma /{\sigma }_{0}\,$> 0.92, the relaxation rate decreases beyond $t$ = 6 mins. Therefore at 1000 °C, a power law fit is applied to obtain a best-fit for the strain rate relaxation, but only for Δ*t* = 3 mins, such that the temporal dependence need not be considered in this time interval. At 950 °C, the strain rate is almost independent of time for any given $\sigma /{\sigma }_{0}$ in the range examined. Therefore, at 950 °C a power law fit can be applied to obtain a best-fit for the experimental data, where the temporal dependence is absent.

It is difficult to fit an Arrhenius-type equation for the relaxation strain rate over the entire temperature interval [950–1000 °C] c.f. Equations 6 and  7. However, since the variation in relaxation rate is less than an order of magnitude over the 50 °C temperature interval for any given $\sigma /{\sigma }_{0}$, as a first approximation the relaxation strain rate can therefore be assumed to be only a function of $\sigma /{\sigma }_{0}$. A simplification to Equation 6 can be thus applied,

10 $${\dot{{\rm{\varepsilon }}}}^{{\rm{rel}}}={A}^{{\rm{rel}}}\exp {(\frac{{\rm{\sigma }}}{{{\rm{\sigma }}}_{0}})}^{{{\rm{n}}}^{{\rm{rel}}}}$$

where the activation energy is included within the pre-exponential factor. Consequently for $\sigma /{\sigma }_{0}$ < 1, the fitting parameters for ${\dot{\varepsilon }}^{{\rm{rel}}}$ are given in Table 2. A comparable stress exponent (n ≈ 10–12) is obtained at both temperatures.

**Table 2:** Fitting parameters of the visco-plasticity model for $\sigma /{\sigma }_{0} < 1$.

T (°C)	${\dot{{\boldsymbol{\varepsilon }}}}^{{\bf{v}}{\bf{p}}}$ (s^−1^)				
	${\dot{{\boldsymbol{\varepsilon }}}}^{{\bf{r}}{\bf{e}}{\bf{l}}}$ (s^−1^)		${\dot{{\boldsymbol{\varepsilon }}}}^{{\bf{c}}{\bf{r}}}$ (s^−1^)		
	${{\boldsymbol{A}}}^{{\bf{r}}{\bf{e}}{\bf{l}}}={{\boldsymbol{A}}}_{0}^{{\bf{r}}{\bf{e}}{\bf{l}}}\,{\bf{e}}{\bf{x}}{\bf{p}}(-\frac{{{\boldsymbol{Q}}}^{{\bf{r}}{\bf{e}}{\bf{l}}}}{{\boldsymbol{RT}}})$ **(s** ^**−1**^ **)**	${{\boldsymbol{n}}}^{{\bf{r}}{\bf{e}}{\bf{l}}}$	${{\boldsymbol{A}}}_{0}^{{\boldsymbol{cr}}}\,$(s^−1^)	${{\boldsymbol{Q}}}^{{\bf{c}}{\bf{r}}}$ (kJ/mol)	${{\boldsymbol{n}}}^{{\bf{c}}{\bf{r}}}$
950 °C	$2\times {10}^{-6}$	10	$5\times {10}^{26}$	773	11
1000 °C	$7\times {10}^{-6}$	12	$5\times {10}^{26}$	773	12

Likewise, the creep strain rate can be directly obtained from the isothermal hold at peak stress prior to relaxation. Figure 6(b) plots the creep strain rate with respect to $\sigma /{\sigma }_{0}$ over the 9 mins isothermal holds at 950 °C and 1000 °C. It can be observed that the creep rate increases with $\sigma /{\sigma }_{0}$. At 1000 °C, the creep strain rate is an order of magnitude greater than that at 950 °C for a given value of $\sigma /{\sigma }_{0}$. Therefore unlike in the case of stress relaxation, the temperature dependence of the creep rate cannot be neglected. Further at both temperatures, the creep strain rate is near-constant, *i.e*. linear plots of creep strain versus temperature in Figure﻿s 6(c and d).

The stress exponent corresponding to the best-fit curves for the creep rates at 950 °C and 1000 °C is n ≈ 11–12. Since the conditions are close to steady-state creep (constant creep rates), we consider the typical activation energies reported for such conditions, where *Q* ~ 750–770 kJ/mol^10^. Accordingly the pre-exponential factor is, *A* ^cr^ ~ 5 × 10^26^ s^-1^. The fitting parameters for ${\dot{\varepsilon }}^{{\rm{cr}}}$ are given in Table 2. It follows therefore that a similar stress exponent is observed both for creep as well as stress relaxation, but lower activation energy is encountered in creep.

**Figure 7:**
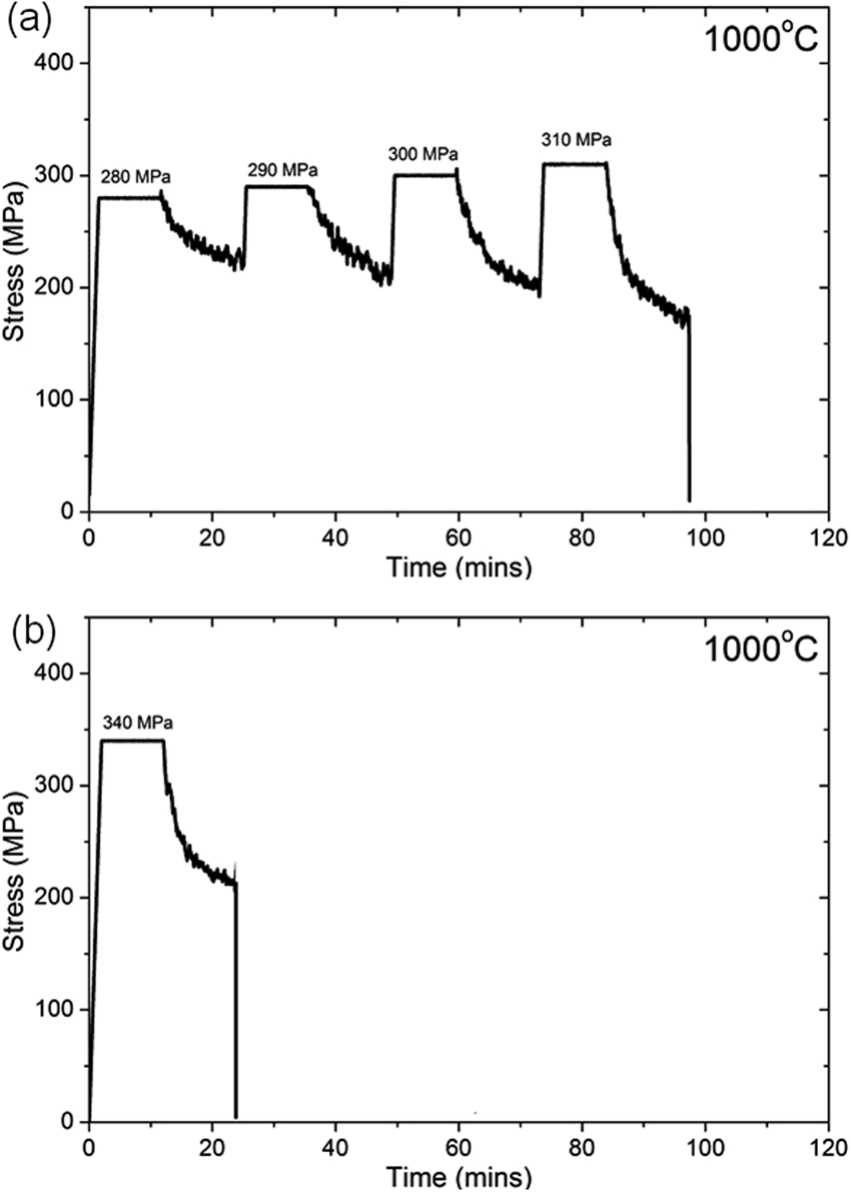
Stress relaxation corresponding to (**a**) cyclically loaded sample with respect to (**b**) single loading experiment.

The implications of prior induced deformation, at stresses close to yield stress on subsequent isothermal stress relaxation tests have been examined at 1000 °C. Figure 7 plots the evolution of stress during stress relaxation cycles at 1000 °C. In the first case, loading is commenced at 280 MPa with an incremental increase in stress of 10 MPa up to 310 MPa. Further at each stress, relaxation up to 9 mins is carried out and in this way the role of prior deformation is incorporated in each subsequent stress relaxation test. Therefore when the applied stress is 310 MPa, the sample has actually been prior loaded and relaxed successively over three previous cycles. In a second experiment the sample is heated to 1000 °C and relaxation commenced on application of a stress of 340 MPa (*i.e*. no prior cyclic loading). It is noted that the initial stress in the ﻿second case exceeded that in the previous case and also exceeded the yield stress. The measured average relaxed stress for both cases over successive time intervals are similar and tabulated in Table 3.

**Table 3:** Comparison of the role of prior induced deformation on stress relaxation during the isothermal relaxation test at 1000 °C.

Applied stress (MPa)	﻿﻿**Δσ﻿** (MPa)		
	0–3 mins	3–6 mins	6–9 mins
310	56	106	121
340	59	101	120

### Modelling of Stress and Strain during Cooling

**Figure 8:**
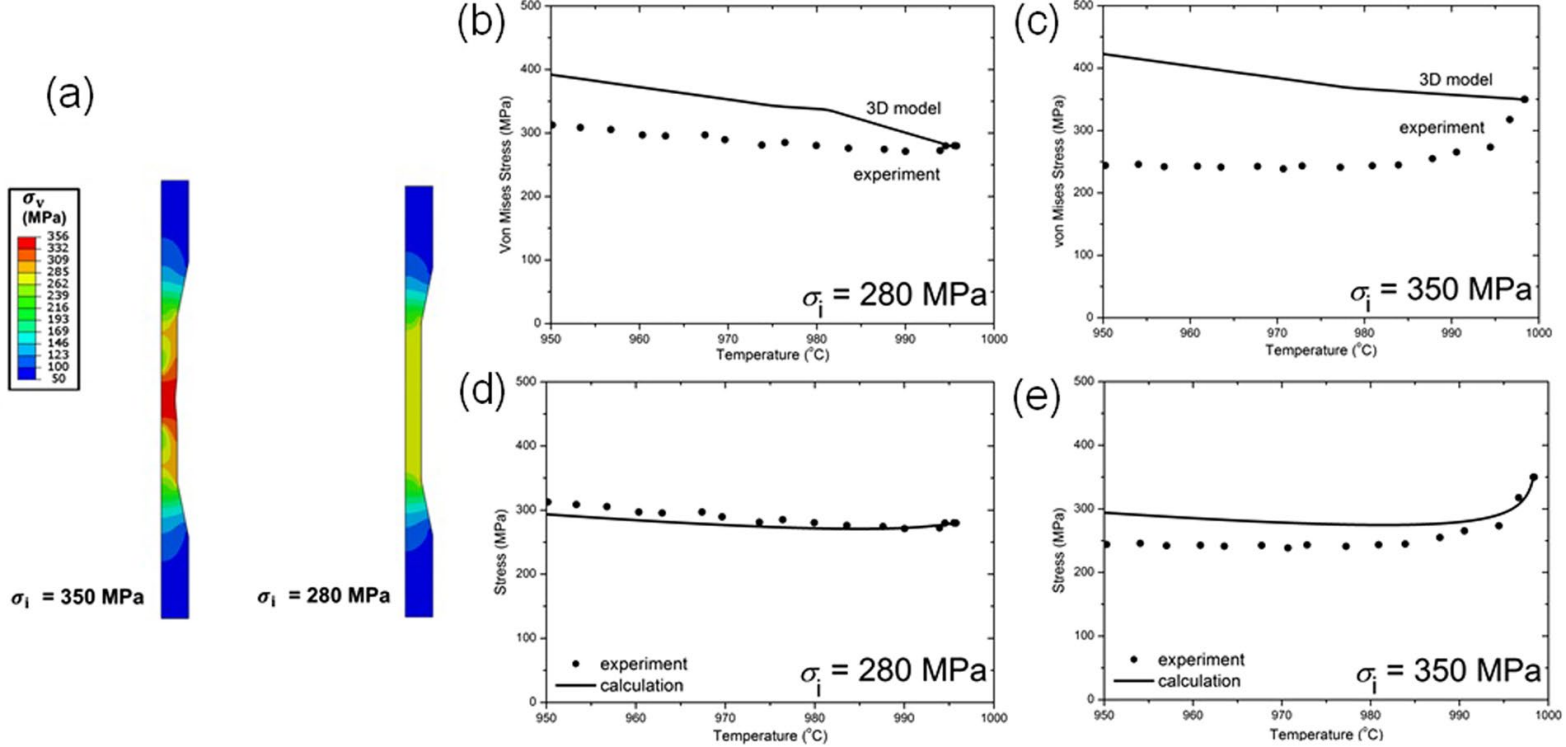
(**a**) The distribution of the predicted stress from 3D Abaqus model captured at 950 °C. Evolution of stress from calculations and juxtaposed experimentally measured stress at (**b**) 280 MPa and (**c**) 350 MPa applied stresses. These are compared with evolution of stress from a 1D model calculation alongside experimentally measured stresses for initial stresses; (**d**) 280 MPa, (**e**) 350 MPa.

Figure 8(a) illustrates the distribution of calculated von Mises stress after cooling from 1000 °C to 950 °C along a vertical cross section through the cylindrical axis of the test samples in expts. 1 and 2. The variation of von Mises stress at the central node of the specimen gauge length with temperature is also shown in Figure﻿s 8(b and c) corresponding to expt. 1 and expt. 2 respectively. The elasto-plastic model indicates that the von Mises stress increases continuously when the temperature decreases from 1000 °C irrespective of the initial stress. The von Mises stress for both the *σ*_i  _= 350 MPa and *σ*_i  _= 280 MPa tests increases by ~70 MPa when cooling between 1000 °C and 950 °C. The kink in the curve at ~980 °C in Figure  8(b) indicates that deformation switches from elastic to plastic deformation upon cooling, while in Figure  8(c) the kink at ~980 °C indicates the onset of elastic deformation on further cooling up to 950 °C. However, this is not consistent with the experimental measurements, where not only is the measured stress lower than the calculated but also in the case of expt. 2, the macroscopic stress decreases sharply during the early stages of cooling [juxtaposed experimental measurements in both Figures  8(b and c)]. This indicates that softening occurs during cooling even at the casting time-scale and requires visco-plasticity such as creep and stress relaxation modes to be considered, which is absent in the elasto-plastic model. The visco-plastic term in Equation 9 can be evaluated by integration of the experimentally determined visco-plastic equation, which becomes even more straightforward when the temporal variation is absent, as in Table 2. Therefore, using a simple 1D model the stresses can be re-calculated using the elasto-viscoplastic approach. Figures  8(d and e) shows the calculated von Mises stress during cooling and also superimposed is the experimentally measured values. The time interval for computation was 1 sec, which is comparable to the data acquisition time for both the extensometer measured strain and load cell measured stress. It can be observed that there is an overall good correlation observed between the experimentally measured as well calculated stresses in expt. 1, but in expt. 2 the modelling predictions still significantly over-estimates the experimentally measured stress.

## Discussion

### Lattice Strains and Deformation Mechanisms during Visco-plasticity

The lattice strains in Figure  2 unlike in ref. 16, include contributions from both elastic loading prior to cooling as well as the misfit stresses that arise from the difference in thermal expansion between the γ and γ′ phases (${\rm{\Delta }}\alpha \,=\,{\alpha }^{\gamma }-\,{\alpha }^{{\gamma }^{^{\prime} }}$). In expt. 2, stress relaxation is accompanied by a decrease in the macro-stress with an accompanying decrease in lattice strain in both γ and γ′. This is a clear evidence of visco-plasticity. Further, the near-constant stress during subsequent cooling down to 950 °C indicates that thermal strain must be primarily accommodated through plastic deformation. On the other hand in expt. 1, when limited stress relaxation occurs in the initial stages and at a later instance during cooling, a similar pattern was observed in both phases, but the magnitude is at least three-fold and eight-fold lower in γ and γ′ phases respectively, c.f. expt. 2. This is because of the lower initial applied stress. However, there is an increase in stress during subsequent cooling down to 950 °C. Since the stress is lower than the yield stress, this indicates that either the deformation is entirely elastic or plastic deformation occurs and is accompanied by work hardening. It is noted that an increase in elastic strain is primarily observed in the γ′ phase.

This aspect is elucidated in the 3D modelling results which are elasto-plastic in nature and in the absence of time-dependent plastic deformation. The calculated stress significantly exceeds the experimentally measured stress. Therefore, time dependent deformation must occur in both the cooling experiments. In expt. 2, a low dislocation density within the γ channels and the extended stacking faults traversing several precipitates was observed when stress relaxation is dominant. While in expt. 1 the stresses are always lower than the yield stress and the high dislocation density must therefore arise during cooling and could be directly attributed to the visco-plastic strain. From the accompanying increase in lattice strain; $\,{\rm{\Delta }}{\varepsilon }^{{\rm{el}}}$ ~ 0.2 × 10^−3^, an approximate increase in the macro-stress can be obtained as, Δ*σ* ~ $\,{\rm{\Delta }}{\varepsilon }^{{\rm{el}}}$ *E* = 20 MPa, where *E* for γ′ in the [100] direction is 85 GPa^26^. This agrees well with the calculated macro-stress at 950 °C in expt. 1. Further, shearing of precipitates by dislocations must imply that the critical resolved shear stress of γ′ has been exceeded from the pile-up of interfacial dislocations in the γ channels, such that they are then able to enter the precipitate and shear them. The parameter that determines the interfacial dislocation network is the misfit parameter^27^. A greater negative misfit that exists in expt. 1 compared with expt. 2, see Fig. 2(c), accounts for the enhanced interfacial dislocation density in expt. 1, as observed in the TEM micrographs. For a negative misfit, the precipitate is in hydrostatic tension and the γ channels in biaxial compression^28^. It has been shown that the resolved shear stress in the vertical and horizontal channels is proportional to the applied stress and the sum of the applied and misfit stresses. A negative misfit therefore increases the stress in the horizontal channels^17^.

When stress relaxation predominates, even with a larger visco-plastic strain, there is a markedly lower interfacial dislocation density in the γ channels and a significantly reduced occu﻿rrence of γ′ precipitate shearing. The smaller negative misfit accounts for a lower interfacial dislocation density. Further, for the dislocations to enter the γ channel, they have to bow into the channels. However, the channel widths are similar in both cases and cannot explain the vastly different dislocation arrangements in expts. 1 and 2. In the case of stress relaxation, the presence of dislocation pairs and dipoles with Burgers vector of $\frac{a}{2}[011]$ indicates that γ′ is sheared through movement of these dislocations consistent with other studies^17^, but few examples of such dislocation pairs were observed, see Figures  5 and 3(e,f). Therefore, some additional mechanism must be invoked to account for the increased visco-plastic strain arising in stress relaxation. In this case, there was the marked presence of long segments/ribbons (>5 µm) of extended stacking faults in the γ phase that traverse several precipitates (Figure  3(d)). Similar observations have been reported in stress relaxation as well as in primary creep, where the principle deformation micromechanism is through creation of extrinsic/intrinsic stacking faults on {111} planes in the precipitate^12, 29, 30^. The stacking fault ribbons are thus able to pass through both phases without a change in the crystal structure. No attempt was made to determine the nature of the partials in our analysis, but suffices to assert that the migration of these observed extended stacking fault ribbons will primarily account for the visco-plastic strain during stress relaxation.

### Implications to Modelling of Stress and Strain during Cooling

In the case of a simple axi-symmetric geometry during directional heat flux, the von Mises stress is greatest along the longitudinal direction, to the first approximation, which reduces the current analysis to a 1D case^9^. The Norton model^28^, which is commonly used to describe visco-plasticity, assumes steady-state creep and the various parameters, stress exponent (*n*), activation energy (*Q*) and pre-exponential factor (*A*), are then determined from a series of creep tests^10^. However, the time period in those creep tests was of the order of hours, unlike in our experiments where from the local cooling rate the time interval for a temperature difference of 3 °C is 3 mins, which is of the order of the local solidification time, and the creep rate was observed to be near-constant. It is therefore reasonable to adopt the Norton-type equation and to determine $n$, *Q* and $A$ *via* curve-fitting. Accordingly, *n* ﻿~ 11–12, which compares well with stress exponents reported for secondary creep in CMSX-4, although these values were reported at lower temperatures (850 °C) and higher stresses (550–750 MPa)^29, 30^. Likewise, $A\,=\,5\,\times \,{10}^{26}$ s^−1^ and $Q$ ~ 773 kJ mol^−1^ corresponds to values that have been reported from long term creep experiments on as-cast microstructures between 1000 °C and 1150 °C in CMSX-4^10^. A good correlation is observed for the evolution of stress with temperature for both calculated and measured values, as in Figure  8(d). This unequivocally demonstrates that visco-plasticity is dominated by creep in expt. 1. Moreover, the time interval for calculation (1 sec) is of the same order as the acquisition time interval for the extensometer strains and stresses. Hence, the creep law derived from the isothermal loading experiments can accurately describe the stress and strain evolution during cooling.

When stress relaxation is dominant in expt. 2, neither the creep law nor the relaxation law deduced from the isothermal loading experiments can satisfactorily account for the stress evolution in the initial stages of cooling, as in Figure  8(e). Moreover, even if both relaxation and creep are assumed to occur simultaneously and the net visco-plastic strain is considered using the two contributions, the calculated stress significantly over-estimates the experimentally measured stress. It follows at higher stresses, when $\sigma /{\sigma }_{0} > 1$, modifications to the dislocation density are likely to occur rapidly. Therefore, the starting microstructure of the sample in any isothermal loading test becomes important. Such considerations are neglected in conventional approaches^10^, where an as-cast sample is simply heated to a given temperature and a relaxation test performed at that given stress and temperature. Therefore, the critical parameter that needs to be considered is a microstructure based parameter, which takes into account the prior induced deformation at a higher temperature. Specifically, the role of prior induced deformation at stresses close to the yield stress on subsequent stress relaxation is adequately demonstrated in Figure  7 and Table 3. The extent of relaxation in a prior loaded and relaxed sample is similar to that measured in a single relaxation experiment even though in the latter case the stress exceeded the yield stress. This definitively corroborates the role of accumulated deformation and therefore prior existing dislocation density. However, since our emphasis is on the derivation of a constitutive law rather than a microstructure based modelling approach, an appropriate way to accommodate such a parameter would be to conduct interrupted cooling experiments commencing at 1000 °C and cooling up to a series of intermediate temperatures within the studied temperature range and subsequently relaxation tests performed on these samples. A possible methodology for such a cooling experiment for stress relaxation in the temperature range Δ*T*_*n*_, would involve sub-division of the temperature/stress interval as in Table 4. In this way, the prior induced visco-plastic strain (and dislocation density) while cooling to that temperature will be captured in the subsequent isothermal relaxation experiment.

**Table 4:** Proposed testing procedure to derive a constitutive law for the cooling experiment taking into account prior induced deformation.

Test step	Initial cooling condition	Intermediate relaxation temperature	Initial stress before relaxation	Decrease in stress from relaxation
1	*T*, *σ*	$T\,-\,{\rm{\Delta }}{T}_{1}$	$\sigma \,-{\rm{\Delta }}\,{\sigma }_{1}$	${\rm{\Delta }}{\sigma ^{\prime} }_{1}$
2	*T*, *σ*	$T\,-{\rm{\Delta }}\,{T}_{2}$ ${\rm{\Delta }}{T}_{2}\, > {\rm{\Delta }}{T}_{1}$	$\sigma \,-\,{\rm{\Delta }}{\sigma }_{2},$	${\rm{\Delta }}{\sigma ^{\prime} }_{2}$
…	…	…	…	…
n	*T*, *σ*	$T\,-\,{\rm{\Delta }}{T}_{n}$ ${\rm{\Delta }}{T}_{n}\, > \,{\rm{\Delta }}{T}_{n-1}$	$\sigma \,-\,{\sigma }_{n},$	${\rm{\Delta }}{\sigma ^{\prime} }_{n}$

Nevertheless it must be reiterated that this modified approach for performing isothermal loading experiments merits specific application only when visco-plasticity is dominant. This can occur not only during stress relaxation, but also arises during cooling at very high temperatures in the vicinity of the solvus temperature which is close to the end of terminal freezing and where creep will dominate (negligible elasticity). Otherwise standard isothermal loading tests, as in the creep experiments will suffice, given that the prior induced deformation is of second order compared with the applied stress and temperature, when visco-plasticity is less dominant.

## Conclusions

1. Visco-plastic deformation during casting is dependent on the stress, cooling rate and constraints imposed on contraction and comprises of creep and stress relaxation.
2. When creep prevails over relaxation, the material progressively work hardens with an increased dislocation density observed in the γ channels and in the vicinity of the γ/γ′ interface. Shearing of γ′ is also observed.
3. When stress relaxation is the dominant mechanism, the material softens. This is accompanied by a marked decrease in dislocation density. Relaxation in lattice strain is accommodated through the migration of extended stacking faults through both the phases.
4. A similar reduction in lattice strain is observed in the γ and γ′ phases during stress relaxation, without any preferential strain partitioning. In creep however, there is no monotonic trend in lattice strain in the γ phase, while a marginal increase is observed in the γ′ precipitates. However, these lattice strain variations are at least three-fold smaller in γ and eight-fold smaller in γ′ than the changes observed during relaxation.
5. Using a 1D model incorporating a visco-plastic law derived from isothermal creep and stress relaxation experiments, a good correlation was shown to exist between the experimentally measured and calculated stresses during cooling, when creep dominates. However, when stress relaxation is dominant, the history dependence of the microstructure from prior induced deformation during cooling must be considered. Without this, the calculations significantly under-estimate the extent of visco-plasticity.
